# Exploring potential treatment opportunities in a head and neck tumor patient with AdCC: A novel germline ERCC2 mutation case report

**DOI:** 10.1097/MD.0000000000041233

**Published:** 2025-01-03

**Authors:** Yuanhao Liu, Tiantian Han, Didi Guo, Dongsheng Chen, Yunqian Li

**Affiliations:** a Department of Neurosurgery, First Hospital of Jilin University, Changchun, Jilin Province, China; b The State Key Laboratory of Neurology and Oncology Drug Development, Jiangsu Simcere Diagnostics Co., Ltd, Nanjing, China; c Department of Medicine, Nanjing Simcere Medical Laboratory Science Co., Ltd., Nanjing, China.

**Keywords:** adenoid cystic carcinoma, chemotherapy, DDR pathway, *ERCC2*, PARPi

## Abstract

**Rationale:**

Adenoid cystic carcinoma (AdCC) is an invasive head and neck malignancy characterized by unpredictable growth, extensive perineural invasion, a high rates of metastasis, and poor survival rates. Genetic alterations, including *MYB-NFIB* and *MYBL1-NFIB* fusions, and mutations within the Notch signaling and DNA damage repair pathways, have been identified.

**Patient concerns:**

A 58-year-old female presented with a space-occupying lesion of the anterior cranial fossa floor during a physical examination and sought further consultation in July 2022.

In our case, a 58-year-old woman was incidentally found to have an anterior cranial fossa lesion during a routine physical examination, which was subsequently confirmed as AdCC following postoperative immunohistochemistry.

**Diagnoses:**

Based on these imaging and histopathological findings, a diagnosis of AdCC was established. Integrating the genetic test results, the case was diagnosed as MYB or MYBL1 fusion-negative AdCC. This case report highlights a rare molecular signature of *ERCC2* and *BRCA2* inactivation in AdCC, in the absence of *MYB* or *MYBL1* fusions.

**Interventions:**

The patient underwent postoperative radiotherapy (RT) to the primary site approximately 2.5 months postsurgery. The concurrent presence of germline ERCC2 and somatic BRCA2 mutations offers novel insights into potential treatment strategies for this rare malignancy.

**Outcomes:**

To date, no recurrence has been observed during follow-up.

**Lessons:**

We found a novel germline *ERCC2* mutation and somatic *BRCA2* mutation in a patient with AdCC. Our findings expand the molecular landscape of rare *MYB* or *MYBL1* fusion-negative AdCC patients and provide a potential therapeutic strategy for this rare head and neck tumor.

## 1. Introduction

Adenoid cystic carcinoma (AdCC) is an invasive carcinoma occurring in the head and neck region, characterized by unpredictable growth, extensive perineural invasion, a high rates of metastasis, and consequently, a low survival rate.^[1]^ Surgical intervention and radiotherapy are the primary treatment strategies for AdCC, with chemotherapy, targeted therapy, and immunotherapy also being utilized to enhance patient survival.^[2]^ Genetically, *MYB-NFIB* and *MYBL1-NFIB* alterations are common molecular event in AdCC, identified in 16% to 100% of cases,^[3]^ with preclinical studies targeting these alterations showing promising results.^[4]^ Furthermore, molecular mutations in Notch signal pathway and DNA damage repair (DDR) pathway have been discovered due to the increased application of whole exome sequencing.^[5]^ AdCC has not yet identified a definitive genetic susceptibility gene. However, germline mutations in *BRCA1/2*^[6]^ and gene involved in DNA double-strand repair^[5]^ may elevate the risk of AdCC. The association between mutations in cancer risk gene and AdCC warrants further investigation. In the era of personalized medicine, identifying comprehensive molecular profiles that can inform treatment decisions is crucial. We report a novel germline *ERCC2* mutation and a somatic *BRCA2* mutation in a patient with AdCC, enriching the mutational landscape of AdCC and offering insights into precision therapy.

## 2. Case presentation

**Figure 1.**
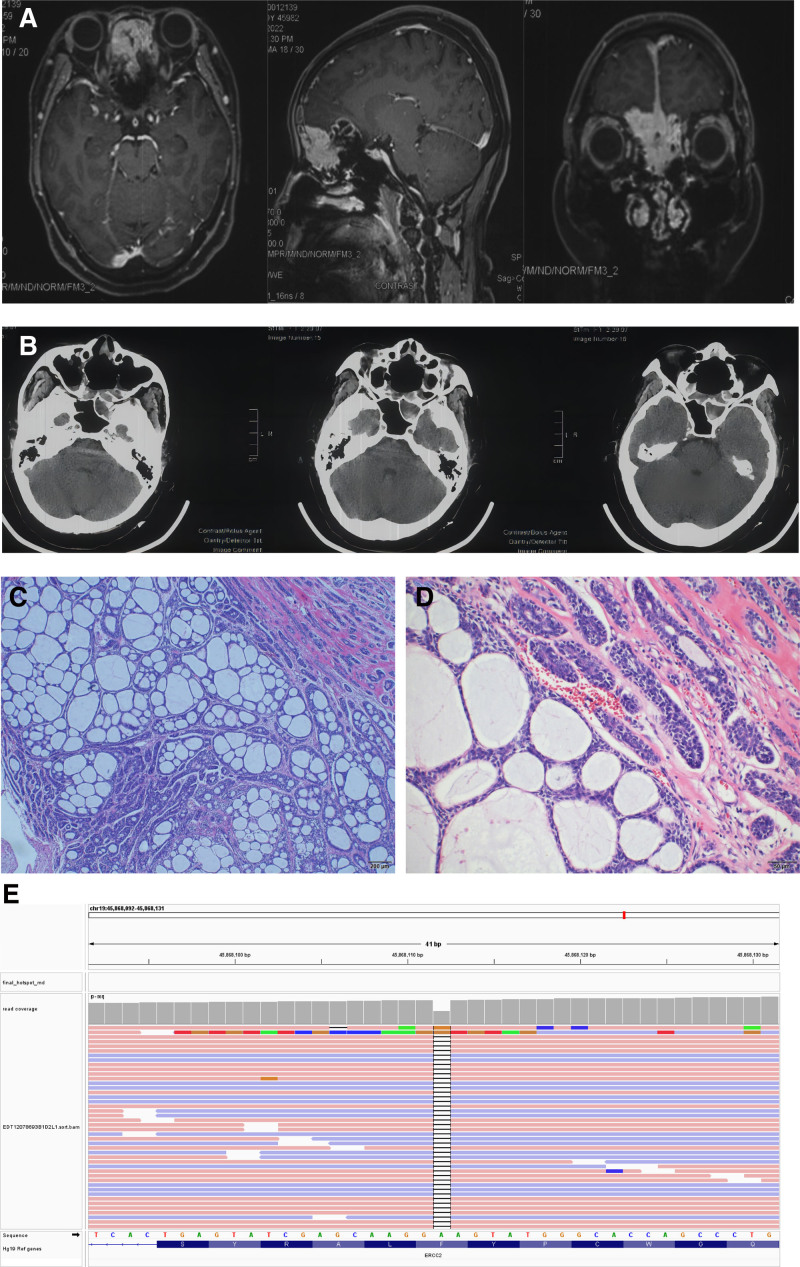
*ERCC2* mutation AdCC in the head and neck. MRI revealed a space-occupying lesion in the preoperative (A) and CT in the postoperative (B). Histopathologic stains (10×) (C) and (100×) (D) from skull base, nasal cavity and cranium tumor tissues suggested AdCC and next-generation sequencing revealed *ERCC2* c.578del (p. F193Sfs*55) mutation (E). AdCC = adenoid cystic carcinoma.

A 58-year-old female presented with a space-occupying lesion of the anterior cranial fossa floor during a physical examination (Fig. 1A) and sought further consultation in July 2022. Subsequently, she underwent intracranial surgery performed under general anesthesia (Fig. 1B). Postoperative immunohistochemistry revealed positive staining for tumor tissues with CK5/6, CK7, CD117, P63, S-100, CKpan, and SMA markers, and negative staining for Syn and GFAP. Additionally, the Ki-67 labeling index in tumor cells was 5%. Based on these imaging and histopathological findings, a diagnosis of AdCC was established (Fig. 1C). The patient underwent postoperative radiotherapy (RT) to the primary site approximately 2.5 months postsurgery. To date, no recurrence has been observed during follow-up.

To identify potential therapeutic targets for reducing the high recurrence rate postsurgery, the patient underwent whole exome sequencing using tumor samples and paired blood samples. Genomic profiling observed potential cancer-diver gene mutations, including a somatic mutation in *BRCA2* c.9097del (p.T3033Lfs*29) with a allelic frequency of 2.37%, and a germline mutation in *ERCC2* c.578del (p.F193Sfs*55) with a allelic frequency of 48.3%, as well as gene amplifications of *MYC* (copy number: 3.27), *CDK4* (copy number: 3.03) in tumor sample. The *ERCC2* mutation c.578del (p. F193Sfs*55) was identified as germline likely pathogenic variant in paired blood samples (Fig. 1D). The c.578del mutation in *ERCC2* gene occured in exon 7 and located within the Helicase ATP-binding domain. The mutations may cause a frameshift of the encoded amino acid at position 193, which may affect protein function (UniProt.org). This variant is absent from population databases, such as gnomAD, and has not been reported in publications. However, other frameshift mutations such as NM_000400.4 (*ERCC2*): c.2006_2007insA (p.Lys671fs; Fig. 1E) are documented as pathogenic in Clinvar (variation ID: 1028729) and loss-of-function variants in *ERCC2* are recognized as pathogenic. Additionally, no *MYB* or *MYBL1* fusions were detected. Our case reveals a rare molecular signature of co-occurring *ERCC2* inactivation and *BRCA2* inactivation in AdCC.

## 3. Discussion

AdCC is a rare, insidious, and highly recurrent head and neck malignancy, with no effective systemic therapy yet identified.^[1]^ The carcinogenic mechanism of AdCC has been gradually elucidated, mainly involving the translocation of *MYB* family genes.^[3]^ Although clinical study reveal no significant difference in overall survival (OS) between AdCC patients with or without *MYB* or *MYBL1* fusion,^[3]^ genomic analysis helps to explore the connection between pathways and target genes in AdCC diagnosis and potential treatments. We report a rare case of AdCC with co-occurring germline *ERCC2* and somatic *BRCA2* mutations and mutually exclusive with *MYB* or *MYBL1* fusion.

The ERCC family contains 4 core tumor-associated genes (*ERCC2, ERCC3, ERCC4, ERCC5*) and the XPD protein encoded by *ERCC2* serves as the helicase subunit of the transcription factor IIH (TFIIH) complex, which is required for DNA damage verification.^[7,8]^ ERCC2 functions as a regulator of the nucleotide excision repair (NER) pathway, which is responsible for repairing bulky DNA lesions induced by environmental mutagens, UV irradiation, and certain chemotherapeutic agents. Acting as an ATP-dependent 5’ to 3’ helicase, ERCC2 unwinds damaged DNA to facilitate access for other NER factors involved in subsequent repair.^[9,10]^ Heterozygous germline mutations in *ERCC2* have been reported in lung adenocarcinoma,^[11]^ dermatofibrosarcoma protuberans.^[12]^ Our case is the first report of an *ERCC2* germline variant in AdCC, and we are not sure whether this mutation is directly related to genetic susceptibility to AdCC, which needs to be supported by more clinical and basic studies, but our case provides a comprehensive molecular characterization for *MYB* or *MYBL1* fusion-negative AdCC.

*BRCA2* has a particularly significant function in homologous recombination-mediated DNA repair. The risk of *ERCC2* LOF mutations with AdCC is not clear, although some relevant cases have been reported.^[5]^ Preclinical and clinical studies have linked *ERCC2* loss of function to cisplatin sensitivity.^[9]^ Studies have reported that pan-cancer patients with DDR dysfunction like *BRCA1/2* or *ERCC* mutations have better OS after immune checkpoint inhibitors treatment.^[13,14]^ For DDR-related mutations, PARP inhibitors may be effective for AdCC, but no reports have been reported so far. Therefore, we have reason to believe that *ERCC2* may be a potential therapeutic target for AdCC.

## 4. Conclusion

We found a novel germline *ERCC2* mutation and somatic *BRCA2* mutation in a patient with AdCC. Our findings expand the molecular landscape of rare *MYB* or *MYBL1* fusion-negative AdCC patients and provide a potential therapeutic strategy for this rare head and neck tumor.

## Author contributions

**Writing – original draft:** Yuanhao Liu, Tiantian Han, Didi Guo.

**Writing – review & editing:** Tiantian Han, Dongsheng Chen, Yunqian Li.

## References

[1] Ho AS Kannan K Roy DM. The mutational landscape of adenoid cystic carcinoma. Nat Genet. 2013;45:791–8. 23685749 10.1038/ng.2643 PMC3708595

[2] Fang Y Peng Z Wang Y. Current opinions on diagnosis and treatment of adenoid cystic carcinoma. Oral Oncol. 2022;130:105945. 35662026 10.1016/j.oraloncology.2022.105945

[3] Persson M Andersson MK Mitani Y. Rearrangements, expression, and clinical significance of MYB and MYBL1 in adenoid cystic carcinoma: a multi-institutional study. Cancers. 2022;14:3691. 35954356 10.3390/cancers14153691 PMC9367430

[4] Drier Y Cotton MJ Williamson KE. An oncogenic MYB feedback loop drives alternate cell fates in adenoid cystic carcinoma. Nat Genet. 2016;48:265–72. 26829750 10.1038/ng.3502 PMC4767593

[5] Ho AS Ochoa A Jayakumaran G. Genetic hallmarks of recurrent/metastatic adenoid cystic carcinoma. J Clin Invest. 2019;129:4276–89. 31483290 10.1172/JCI128227 PMC6763222

[6] Shen TK Teknos TN Toland AE Senter L Nagy R. Salivary gland cancer in BRCA-positive families: a retrospective review. JAMA Otolaryngol Head Neck Surg. 2014;140:1213–7. 25257187 10.1001/jamaoto.2014.1998

[7] Kobayashi T Kuraoka I Saijo M. Mutations in the XPD gene leading to xeroderma pigmentosum symptoms. Hum Mutat. 1997;9:322–31. 9101292 10.1002/(SICI)1098-1004(1997)9:4<322::AID-HUMU4>3.0.CO;2-7

[8] Tapias A Auriol J Forget D. Ordered conformational changes in damaged DNA induced by nucleotide excision repair factors. J Biol Chem. 2004;279:19074–83. 14981083 10.1074/jbc.M312611200 PMC4494833

[9] Liu D Plimack ER Hoffman-Censits J. Clinical validation of chemotherapy response biomarker ERCC2 in muscle-invasive urothelial bladder carcinoma. JAMA Oncol. 2016;2:1094–6. 27310333 10.1001/jamaoncol.2016.1056 PMC5515075

[10] Evans E Moggs JG Hwang JR Egly JM Wood RD. Mechanism of open complex and dual incision formation by human nucleotide excision repair factors. EMBO J. 1997;16:6559–73. 9351836 10.1093/emboj/16.21.6559 PMC1170260

[11] Liu L Cui J Liu S Pan E Sun L. Case report: lung adenocarcinoma associated with germline ERCC2 frameshift mutation. Front Oncol. 2023;13:1177942. 37223679 10.3389/fonc.2023.1177942 PMC10200934

[12] Zhang Q Ju Y You X Sun T Ding Y. Case report: Identification of a novel heterozygous germline ERCC2 mutation in a patient with dermatofibrosarcoma protuberans. Front Oncol. 2022;12:966020. 36033485 10.3389/fonc.2022.966020 PMC9399496

[13] Shi C Qin K Lin A. The role of DNA damage repair (DDR) system in response to immune checkpoint inhibitor (ICI) therapy. J Exp Clin Cancer Res. 2022;41:268. 36071479 10.1186/s13046-022-02469-0 PMC9450390

[14] Chen C Liu H Li Y Liu J. Association of ERCC family mutations with prognosis and immune checkpoint inhibitors response in multiple cancers. Sci Rep. 2023;13:13925. 37626083 10.1038/s41598-023-40185-7 PMC10457344

